# Imaging Inflammation with Positron Emission Tomography

**DOI:** 10.3390/biomedicines9020212

**Published:** 2021-02-19

**Authors:** Janette Iking, Magdalena Staniszewska, Lukas Kessler, Jasmin M. Klose, Katharina Lückerath, Wolfgang P. Fendler, Ken Herrmann, Christoph Rischpler

**Affiliations:** 1 Department of Nuclear Medicine, University of Duisburg-Essen and German Cancer Consortium (DKTK)-University Hospital Essen, Hufelandstraße 55, D-45147 Essen, Germany; Magdalena.Staniszewska@uk-essen.de (M.S.); Lukas.Kessler@uk-essen.de (L.K.); Jasmin.Klose@uk-essen.de (J.M.K.); Wolfgang.Fendler@uk-essen.de (W.P.F.); Ken.Herrmann@uk-essen.de (K.H.); Christoph.Rischpler@uk-essen.de (C.R.); 2 Department of Molecular and Medical Pharmacology, David Geffen School of Medicine, University of California Los Angeles, 650 Charles E young Drive S, Los Angeles, CA 90095, USA; KLueckerath@mednet.ucla.edu

**Keywords:** molecular imaging, inflammation, PET, FDG, SSTR, FAPI, CXCR4, CCR2, TSPO, integrin

## Abstract

The impact of inflammation on the outcome of many medical conditions such as cardiovascular diseases, neurological disorders, infections, cancer, and autoimmune diseases has been widely acknowledged. However, in contrast to neurological, oncologic, and cardiovascular disorders, imaging plays a minor role in research and management of inflammation. Imaging can provide insights into individual and temporospatial biology and grade of inflammation which can be of diagnostic, therapeutic, and prognostic value. There is therefore an urgent need to evaluate and understand current approaches and potential applications for imaging of inflammation. This review discusses radiotracers for positron emission tomography (PET) that have been used to image inflammation in cardiovascular diseases and other inflammatory conditions with a special emphasis on radiotracers that have already been successfully applied in clinical settings.

## 1. Introduction

Inflammation is a fundamental and well-balanced physiological process necessary for wound healing, protection against pathogens, and tissue homeostasis. Restrained or excessive inflammation, however, can have detrimental effects leading to pathological alterations that can worsen the outcome of patients or even form the basis of the disease itself. Consequently, the immune system and its response to pathological changes play a major role in virtually all diseases ranging from bacterial or viral infectious diseases, neurological disorders, cancer, autoimmune diseases, and cardiovascular diseases. 

The adaptability of the human immune system is one of the reasons why it can react effectively and rapidly against pathogens; at the same time, it may render many novel therapies targeting inflammation or involving the immune system effective in some patients whereas other patients with the same condition do not respond at all. Accordingly, the immune response is being understood as a very individual process that demands customized therapies. Because inflammation is a very dynamic process that involves many immune cell subtypes, it can be challenging to identify the appropriate molecular target and timing for optimal intervention. In this context, molecular imaging has emerged as a helpful research tool to non-invasively visualize and study inflammation in vivo in a variety of diseases especially in a preclinical setting. However, molecular imaging may also provide insight into the individual biology of inflammation which can have diagnostic, therapeutic, and prognostic value for patients.

In recent years, many novel radiotracers and newly developed protocols for inflammation imaging have been particularly applied in the field of nuclear cardiology. This review aims to summarize and discuss radiotracers for positron emission tomography (PET) that have been used to image inflammation in cardiovascular diseases and other inflammatory conditions. Special emphasis is put on tracers that have already been successfully applied in the clinics (Table 1).

## 2. Imaging Targets of Inflammation

### 2.1. Carbohydrate Metabolism

#### 2.1.1. Glucose Metabolism

**Figure 1:**
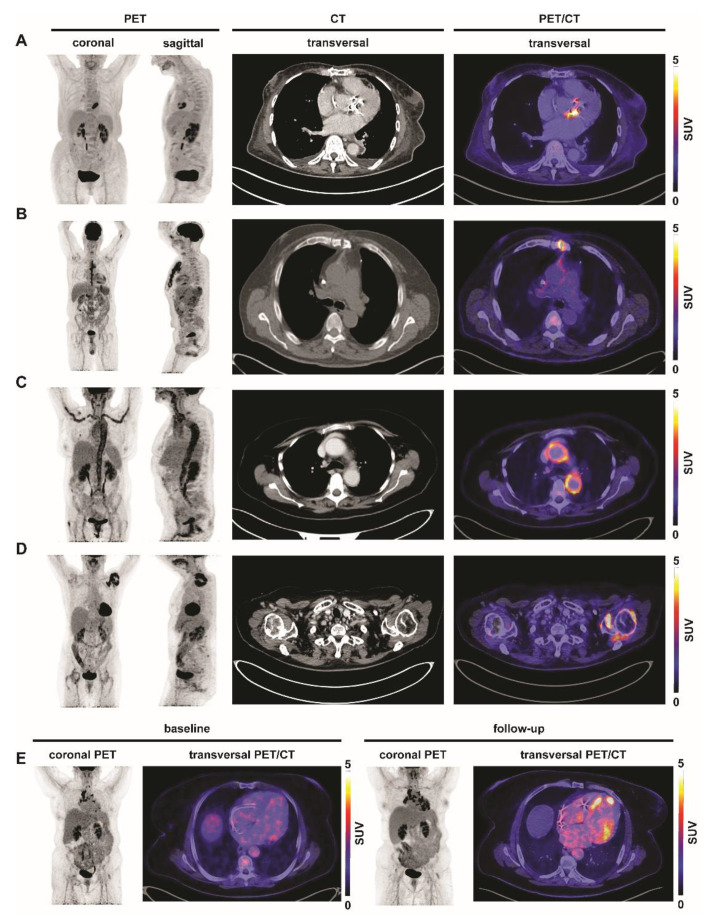
Inflammation imaging with ^18^F-FDG in various inflammatory conditions. Representative coronal, sagittal and transversal ^18^F-FDG PET/CT images of patients with different inflammatory conditions. (**A**) Aortic valve abscess. Patient received aortic valve replacement. Echocardiographic suspicion for infected aortic valve was confirmed by ^18^F-FDG-PET after dietary preparation which revealed circular tracer uptake around the aortic valve reported as aortic valve abscess. (**B**) Fever of unknown origin – osteomyelitis. Patient with fever of unknown origin who underwent sternotomy three months prior imaging. ^18^F-FDG uptake can be seen along the sternum supporting clinical suspicion of osteomyelitis. (**C**) Fever of unknown origin—arteritis. Patient with recurrent fever of unknown origin for three months and increasing c-reactive protein levels. ^18^F-FDG-PET shows inflammation of the large vessels consistent with a Takayasu’s arteritis. (**D**) Polyarthritis. Follow-up ^18^F-FDG-PET of a patient with malignant melanoma and polyarthritis with progressive pain. PET shows active arthritis in the left shoulder. (**E**) Sarcoidosis. Patient with suspicion of progressive sarcoidosis and newly diagnosed cardiac arrhythmia. ^18^F-FDG-PET after dietary preparation show increased uptake in bihilary lymph nodes at baseline. Follow-up ^18^F-FDG PET/CT shows increased inflammatory activity in bihilary lymph nodes and further myocardial involvement. SUV, standardized uptake value.

The most widely used and best-described radiotracer for PET imaging is fluor-18-labelled fluorodeoxyglucose (^18^F-FDG). FDG is a glucose analogue that is primarily taken up by high-glucose-consuming cells such as neurons, brown adipocytes, cardiomyocytes, kidney cells, cancer cells, and inflammatory cells. It is rapidly transported via glucose transporters (GLUTs) into the cytosol, phosphorylated by hexokinase, and intracellularly trapped as FDG-6-phosphate. Phosphorylation by hexokinase prevents ^18^F-FDG from being metabolized until radioactive decay: ^18^F-FDG-6-phosphate cannot be metabolized due to a missing 2-hydroxyl group that is needed for further glycolysis. After radioactive decay, however, the molecule is converted to glucose-6-phosphate which allows it to be metabolized in the same way as normal glucose [68]. Thus, FDG accumulation de- pends on the rate of transport into the cytosol, the enzymatic activity of hexokinase, and the rate of dephosphorylation in the tissue [69]. To date, 14 GLUT have been discovered but only for a few of them the primary physiological substrate is known [70]. The transport of FDG across cell membranes is mediated by GLUT-1 to GLUT-5, but especially significantly elevated expression levels of GLUT-1 and GLUT-3 are considered to be a factor contributing to the accumulation of FDG [69,71]. ^18^F-FDG has been proposed for imaging inflammation and infection partly because ^18^F-FDG uptake has been observed at sites of inflammation during routine imaging of cancer patients. Further studies demonstrated that cells involved in infection and inflammation, especially phagocytes such as neutrophils and monocytes/macrophages, are able to express high levels of glucose transporters, especially GLUT-1 and GLUT-3, as well as hexokinase activity [69,72,73]. Since then, the diagnostic and prognostic value of ^18^F-FDG PET/computed tomography (CT) for imaging infectious and inflammatory diseases has increasingly been acknowledged. It has proven its diagnostic and prognostic worth in several inflammatory conditions including myocardial infarction [1], fever of unknown origin [74], or large-vessel inflammation (Figure 1). However, different diseases might require different imaging protocols; an international consensus on ^18^F-FDG inflammation imaging should therefore be determined in the near future. The nonspecific characteristics of ^18^F-FDG are beneficial and disadvantageous at the same time: on the one hand it allows detection of unknown origins of infection and inflammation with high sensitivity and has a high negative predictive value regarding inflammatory or malignant disease [75]; on the other hand the cellular source of the signal cannot be determined with certainty although it has been shown in vitro that ^18^F-FDG accumulates markedly higher in pro-inflammatory M1 macrophages as compared to reparative M2 macrophages [76]. Due to the unspecific uptake of ^18^F-FDG in various high-glucose consuming cells uptake suppression strategies are often necessary to unmask the potential leukocyte signal. Protocols such as fasting, administration of unfractionated heparin, and high-fat meals which promote fatty acid metabolism instead of glucose uptake have been shown to be effective [77,78].

Uptake suppression strategies are especially important for successful imaging of cardiac inflammation due to high uptake of ^18^F-FDG in cardiomyocytes. For animal studies, ketamine-xylazine anaesthesia has been proven effective for suppression of the ^18^F-FDG signal in cardiomyocytes since it interferes with pancreatic glucose sensing, thereby blocking insulin-dependent glucose transport [77]. Myocardial infarction (MI) initiates a strong immune response that can be imaged successfully with ^18^F-FDG PET. Peak uptake of ^18^F-FDG within the ischaemic heart occurs approximately 3–5 days post-MI and declines over 7–14 days; this is in line with the time course of accumulation of pro-inflammatory M1-like macrophages and CD11b-positive monocytes [79,80,81]. ^18^F-FDG PET combined with magnetic resonance imaging (MRI) has confirmed the biphasic nature of inflammatory and reparative monocyte infiltration into the infarcted heart in a murine model as well as in patients with acute MI [79,82]. Intriguingly, Rischpler and colleagues showed that ^18^F-FDG uptake 5 days after percutaneous coronary intervention (PCI) in patients with MI had an inverse correlation with the cardiac functional outcome measured by MRI 6–9 months post-MI, demonstrating that ^18^F-FDG imaging can serve as a prognostic biomarker for MI [1].

^18^F-FDG PET/CT has also led to advances in other fields of cardiovascular research such as atherosclerosis [3], (cardiac) sarcoidosis [4], and endocarditis [5]. Early imaging studies of atherosclerosis showed that the highest ^18^F-FDG uptake was seen in symptomatic plaques of the carotid arteries indicating that ^18^F-FDG might allow to assess the vulnerability of a plaque [83]. Furthermore, ^18^F-FDG PET was useful for monitoring atherosclerotic activity in the dal-PLAQUE study in which dalcetrabib treatment in combination with low-density lipoprotein-lowering therapy led to a reduction of ^18^F-FDG uptake in the carotid arteries [84]. In cardiac sarcoidosis, ^18^F-FDG PET was shown to respond to immunosuppressive therapy [4,85]. In addition, ^18^F-FDG has been proven useful in other inflammatory conditions such as the detection of aortic valve prosthetic infections (Figure 1A), detection of osteomyelitis (Figure 1B), large-vessel inflammation (Figure 1C), polyarthritis (Figure 1D), or monitoring the progression of sarcoidosis (Figure 1E).

Nonetheless, ^18^F-FDG PET has certain limitations. The unknown cellular source of the ^18^F-FDG signal and the hereof resulting non-specificity, as well as its dependency on glucose levels and renal function limits its use in many clinical scenarios for instance in diabetic patients. Concomitant treatments such as steroids or statins can influence the ^18^F-FDG signal making the assessment of inflammatory activity in some patient populations a difficult endeavor [86,87]. Uptake suppression strategies are necessary especially for cardiac inflammation imaging due to increased ^18^F-FDG uptake in cardiomyocytes [77,78]. Moreover, surgical adhesives used for example for implantations of prosthetic valves can be a factor for false-positive interpretations of prosthetic valve endocarditis [88]. Therefore, other radiotracers have been developed and evaluated for their use in imaging inflammation.

#### 2.1.2. Mannose Receptor

The mannose receptor (CD206) is an interesting imaging target of carbohydrate metabolism. It is mainly expressed by macrophages and immature dendritic cells but also by liver sinusoidal endothelial cells [89]. Interestingly, CD206 is predominantly expressed by M2-like reparative macrophages and therefore serves as a marker to distinguish M1 from M2 macrophages [9,90]. Mannose receptor imaging has been mainly utilized in preclinical oncological and cardiovascular studies (e.g., in atherosclerosis research), most likely because it is overexpressed in unstable high-risk atherosclerotic plaques [9,90]. ^18^F-labelled mannose (^18^F-fluoro-D-mannose; ^18^F-FDM) has been successfully used for imaging inflammation in a rabbit model of atherosclerosis; gallium-68-labelled mannosylated human serum albumin (MSA) has been utilized for visualizing inflammation in myocarditis and atherosclerotic plaques, respectively, in several animal models [9,10,11]. More recently feasibility of imaging atherosclerotic plaques with a ^68^Ga-labelled anti-CD206-nanobody has been shown in an apoE knock-out mouse model [91]. However, correlation of the mannose-directed PET signal with reparative leukocytes and its distinction from that of ^18^F-FDG remains to be determined, and their potential for translation requires further investigation.

### 2.2. Chemokine Receptors

#### 2.2.1. C-X-C Motif Chemokine Receptor 4 (CXCR4)

Chemokines are involved in the recruitment of leukocytes and are thus an attractive target to image inflammation. Chemokine receptor type 4 (CXCR4) and its specific ligand stromal cell-derived factor 1 (SDF1, also known as CXCL12) mediate the migration and recruitment of leukocytes to the site of inflammation and have been proposed as therapeutic targets to modulate inflammation [81,92]. CXCR4 is expressed on several pro-inflammatory immune cell types (monocytes/macrophages, T and B lymphocytes, and/or their progenitor cells) with a pronounced overexpression on macrophages and T lymphocytes [93]. It can be targeted by CXCR4-directed PET tracers such as ^68^Ga-pentixafor, a radiotracer originally developed for cancer imaging [20]. These radiotracers are not being used routinely in the clinic yet but preclinical and clinical pilot studies have shown promising results.

**Figure 2:**
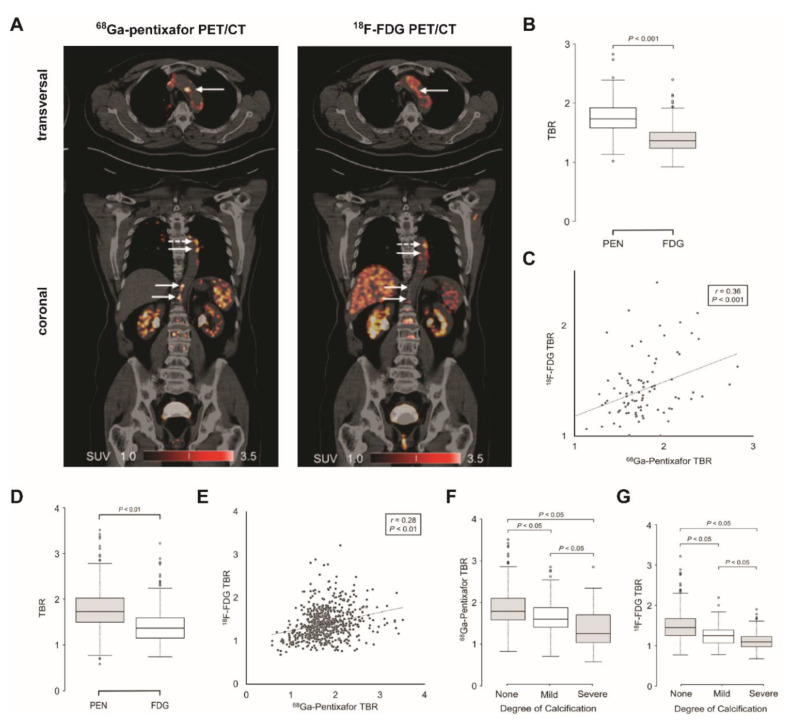
Inflammation imaging of atherosclerosis with ^68^Ga-pentixafor (CXCR4) and ^18^F-FDG. (**A**) Representative transversal and coronal ^68^Ga-pentixafor PET/CT and ^18^F-FDG PET/CT images of patient with multiple myeloma. White arrows point to lesions that differ in uptake of ^68^Ga-pentixafor and ^18^F-FDG. (**B**,**C**) Comparison of ^68^Ga-pentixafor and ^18^F-FDG uptake on per-patient basis. Box plot (**B**) and scatterplot (**C**) showing correlation of mean ^18^F-FDG and ^68^Ga-pentixafor (PEN) uptake as measured by TBR on per-patient basis. (**D**,**E**) Comparison of ^68^Ga-pentixafor and ^18^F-FDG uptake on lesion-to-lesion basis. Box plot (**D**) and scatterplot (**E**) showing correlation of ^18^F-FDG and ^68^Ga-pentixafor (PEN) uptake as measured by TBR on per-lesion basis. (**F**,**G**) ^68^Ga-pentixafor (**F**) and ^18^F-FDG (**G**) uptake in correlation to degree of calcification on lesion-to-lesion basis. Lesions were categorized by calcification degree as noncalcified (˂130 HU), mildly calcified (130–399 HU), or severely calcified (>400 HU), respectively. TBR of ^68^Ga-pentixafor and ^18^F-FDG are lowest in severely calcified lesions and highest in noncalcified lesions. SUV, standardized uptake value; TBR, target-to-background ratio. This research was originally published in JNM.^22^ Kircher M et al. Imaging Inflammation in Atherosclerosis with CXCR4-Directed 68Ga-Pentixafor PET/CT: Correlation with 18F-FDG PET/CT. J Nucl Med. 2020;61(5):751–756. © SNMMI.

In a rabbit model of atherosclerosis, it was shown that macrophage-rich plaques overexpress CXCR4 which can be imaged PET with ^68^Ga-pentixafor [21]. This was confirmed in a study of 72 patients with lymphoma; the study visualized inflamed carotid plaques with ^68^Ga-pentixafor PET/MR imaging. The authors further presented histological evidence for a colocalization of CXCR4 and CD68, a marker for monocytes/macrophages, as well as CD3, a marker for T lymphocytes, in human excised carotid plaques [24]. ^68^Ga-pentixafor was also used to detect vascular inflammation after stent-based reperfusion in 37 patients with acute MI with highest tracer uptake in the culprit lesions [23]. Li and colleagues proposed CXCR4 as an interesting theranostic target for atherosclerosis since CXCR4-directed endoradiotherapy with ^177^Lu-/^90^Y-pentixather for hematologic malignancies showed an anti-inflammatory effect on atherosclerotic plaques [25]. In a recently published study, Kircher et al. suggested superiority of ^68^Ga-pentixafor over ^18^F-FDG imaging for detection and monitoring of atherosclerosis in a study of 92 patients that underwent PET imaging with the two radiotracers for oncological staging. ^68^Ga-pentixafor identified more lesions than ^18^F-FDG, with only a weak correlation between the radiotracers (Figure 2) [22].

In the setting of MI, several pilot studies reported promising results. CXCR4-directed PET imaging showed high tracer uptake in the infarcted myocardium [26,27]. In a murine model of MI, uptake of ^68^Ga-pentixafor in the infarcted myocardium was proportional to leukocyte infiltration as detected by flow cytometry. The authors showed that at 4–6 days post-MI, patients exhibited heterogeneous patterns of CXCR4 expression; this suggests an individual modulation of the chemokine response which could be exploited to select patients for therapeutic intervention [28]. In a small study including 22 patients with acute MI, Reiter and colleagues reported a correlation of high CXCR4 expression in the infarcted myocardium to smaller scar volumes as well as a correlation between splenic uptake of ^68^Ga-pentixafor and change in ejection fraction at follow-up, indicating a possible role for ^68^Ga-pentixafor as an imaging biomarker [29]. However, besides the relatively small patient number, this study was limited by its varying imaging and follow-up time points as well as lacking data on CXCR4/CXCL12 levels to determine endpoints of molecular inflammation. Moreover, the findings of this study are in contradiction to two recently published studies suggesting beneficial effects of CXCR4 blockade in mice with MI [30,31]. The more recent one of these studies reported that the timing of CXCR4 blockade is important and that CXCR4-directed PET imaging can serve as a tool for therapeutic guidance to determine the right timing of treatment. Hess and colleagues further showed a correlation of CXCR4 expression in acute MI patients with cardiac outcome and inflammation parameters suggesting prognostic value of ^68^Ga-pentixafor PET [31].

In non-cardiovascular settings, CXCR4-directed PET has mainly been used for imaging infectious diseases such as osteomyelitis [32] or urinary tract infections [33]. Bouter and colleagues showed that ^68^Ga-pentixafor is superior regarding its diagnostic accuracy in chronic bone infections over technetium-99m(^99m^Tc)-labelled leukocytes and granulocyte-directed ^99m^Tc-besilesomab and comparable to ^18^F-FDG PET [32]. Although CXCR4 PET imaging seems to be one of the most promising preclinical candidates for molecular imaging of inflammation, further studies determining the cellular source of ^68^Ga-pentixafor and controlled prospective trials with more patients in different inflammatory settings are needed to determine the value of CXCR4 imaging for prognosis and therapeutic guidance. Novel tracers with theranostic properties and enhanced CXCR4 affinity are currently being investigated and might help to further improve CXCR4 inflammation imaging [94].

#### 2.2.2. C-C Motif Chemokine Receptor 2 (CCR2)

Another major chemokine receptor that has been targeted for imaging inflammation is the C-C chemokine receptor type 2 (CCR2). CCR2 is highly expressed on infiltrating inflammatory monocytes/macrophages, natural killer cells, and T cells but also on dendritic cells. Interaction of CCR2 with its ligand CCL2 is essential for inducing monocyte release from the bone marrow and their migration into tissues [95,96,97]. However, an imbalance in favor of highly inflammatory CCR2^+^ macrophages can lead to pathological alterations contributing to postinfarction heart failure or resulting in inflammatory conditions such as atherosclerosis and rheumatoid arthritis (RA) [98,99,100].

Liu and colleagues have developed the CCR2 ligand “extracellular loop 1 inverso” (ECL1i) which allosterically binds to the extracellular domain of CCR2 and can be coupled to gallium-68 or copper-64 via DOTA [34]. ^64^Cu-labelled ECL1i was used in a murine model of lung inflammation and in human biopsies from patients with chronic obstructive pulmonary disease (COPD) to visualize CCR2^+^ cells in both species [34]. In another study, the authors demonstrated that ^68^Ga-DOTA-ECL1i uptake was localized to sites of cardiac injury visualized by PET and ex vivo autoradiography [35]. They could further show that tracer uptake was associated with CCR2^+^ monocyte infiltration into the heart. ^68^Ga-DOTA-ECL1i uptake was predictive of both left ventricle (LV) ejection fraction and akinetic area, suggesting a prognostic value of the radiotracer. Ex vivo autoradiography of human heart failure specimens demonstrated binding of the tracer to human CCR2 adding an additional translational value to the study [35]. The authors later compared ^68^Ga- to ^64^Cu-labelled ECL1i in cardiac injury and found comparable radiotracer uptake in the ischemic area [101]. Recently, the group also reported proof-of-concept for ^64^Cu-DOTA-ECL1i PET in the detection of abdominal aortic aneurysm (AAA) [36] and pulmonary fibrosis [37] in rodent models and human tissue. Remarkably, ^64^Cu-DOTA-ECL1i uptake was twice as high in AAA that subsequently ruptured compared to non-ruptured AAA. In addition, ^64^Cu-DOTA-ECL1i uptake decreased in a mouse model of idiopathic pulmonary fibrosis (IPF) after treatment with interleukin-1β blockade or antifibrotic pirfenidone suggesting that CCR2-directed PET can be a useful tool for therapeutic monitoring.

Recently, Wagner and colleagues synthetized the first small-molecule, nonpeptidic, fluorine-18-labelled CCR2-targeting radiotracer. The authors reported an exceptional target affinity and selectivity; however, further in vivo biodistribution and proof-of-concept studies are necessary to support a role for this novel tracer in the setting of inflammation imaging [102].

Despite these promising results, especially regarding prognostic and therapy-monitoring abilities, CCR2-directed PET is still limited by its unspecific cellular source as various inflammatory cells express CCR2. Furthermore, toxicity and biodistribution need to be examined with caution for a safe translation into the clinic. Currently, ^64^Cu-DOTA-ECL1i is being investigated in several phase I clinical trials, amongst others for imaging atherosclerosis (NCT04537403) and lung inflammation (NCT03492762).

### 2.3. Somatostatin Receptors

Somatostatin is a small neuropeptide associated with the post-synaptic response to N-methyl-D-aspartate (NMDA) receptor activation. Somatostatin receptors (SSTRs) are G protein-coupled receptors that are important diagnostic and therapeutic targets in oncology especially for the management of neuroendocrine tumors and meningiomas [103]. SSTRs, in particular the receptor subtype SSTR_2A_, are highly expressed on activated pro-inflammatory M1-like macrophages with almost no expression on monocytes, T or B lymphocytes, natural killer cells, platelets, neutrophils, or endothelial cells; this indicates that SSTR_2_ may offer improved cell specificity as an imaging target for inflammation compared to glucose metabolism [13]. It can be targeted using synthetic somatostatin analogues, such as DOTATOC, DOTANOC, and DOTATATE, which mainly differ in their affinity to the five receptor subtypes (SSTR_1-5_). DOTATATE shows the highest affinity to SSTR_2_, potentially making it the best SSTR targeted radiotracer for inflammation imaging by PET [104].

Numerous studies have successfully utilized ^68^Ga-labelled DOTA-peptides for PET inflammation imaging. Many of these studies originated from cardiovascular research possibly due to higher cell specificity and improved signal-to-background-ratio of DOTA-peptides compared to ^18^F-FDG imaging. In a prospective observational trial, Tarkin et al. compared ^18^F-FDG and ^68^Ga-DOTATATE PET in 42 patients with atherosclerosis and found that DOTATATE imaging was superior in coronary plaque imaging, specific uptake into macrophages, and in discriminating high-risk versus low-risk coronary lesions [13]. In a following substudy, they demonstrated that SSTR-directed PET detected residual postinfarction cardiac inflammation both in patients with recent MI (less than 3 months) as well as in older ischemic injuries [14]. Malmberg and colleagues showed (in 60 oncological patients) that uptake of ^64^Cu-DOTATATE, but not of ^68^Ga-DOTATOC, was correlated with cardiovascular risk factors, further supporting the hypothesis that DOTATATE might be the best suited SSTR-directed tracer for PET inflammation imaging [105].

SSTR-directed PET was also shown to be superior over ^67^Ga-scintigraphy for identification of muscle, lymph node, and uvea lesions in a study including 20 patients with sarcoidosis [15]. Subsequent pilot studies examining cardiac sarcoidosis confirmed superiority of SSTR-directed PET vs. ^18^F-FDG PET as well as a close correlation of results from cardiac magnetic resonance imaging (CMR) and SSTR-directed PET/CT [16,17]. The latter was confirmed for other sources of myocardial inflammation such as pericarditis, myocarditis, and MI [18].

Additionally, SSTR-directed PET or scintigraphy was studied in several other inflammatory conditions such as idiopathic pulmonary fibrosis, histiocytosis, tuberculosis, cardiac allograft rejection, and small vessel vasculitis that cannot be extensively covered in this review. For a comprehensive overview over the current literature and most promising candidates for somatostatin receptor imaging by single photon emission computed tomography (SPECT) and PET in patients with chronic inflammatory disorders, please refer to Anzola and colleagues [19].

As promising as SSTR-directed PET for inflammation imaging may seem, there are still some obstacles that need to be overcome in the future. Implementation of ^68^Ga-labelled DOTA-peptides in routine clinical practice, for instance, is often limited by the need of an on-site ^68^Ge/^68^Ga generator [104]. Furthermore, SSTR-directed PET might display a lack of sensitivity in inflammation that is not macrophage-driven such as chronic inflammation. Further prospective clinical studies are needed to determine the prognostic value of the imaging signal and to clarify the binding profile of the used radionuclides. Lastly, clear imaging protocols for SSTR-directed PET imaging of inflammation are needed to ensure reproducibility and standardization.

### 2.4. Cell Adhesion Molecules (CAMs)

CAMs particularly integrins are crucially important in mediating processes such as immune cell trafficking into tissues, effector cell activation and proliferation, the formation of the immunological synapse between immune cells or between immune cell and the target cell, both during homeostasis and during inflammation and cancer [106].

The transmembrane receptor alpha-v beta-3 integrin (α_v_β_3_) mediates cell adhesion and plays a vital role in angiogenesis, making it a compelling imaging target in many oncologic and inflammatory conditions [107]. Imaging probes containing arginylglycylaspartic acid (RGD)—the most common peptide motif responsible for cell adhesion to the extracellular matrix—such as ^18^F-galacto-RGD, ^68^Ga-PRGD2, and ^18^F-fluciclatide bind to α_v_β_3_ with high affinity and allow noninvasive visualization of α_v_β_3_ expression in vivo [108]. In atherosclerosis, α_v_β_3_ integrin may be directly involved in the degradation of the protective fibrous cap of atherosclerotic plaques making it an interesting target to assess plaque vulnerability. In a study of 10 patients with high-grade carotid artery stenosis, ^18^F-galacto-RGD uptake was significantly higher in atherosclerotic plaques than in nonstenotic areas. Radiotracer uptake significantly correlated with α_v_β_3_ expression within the plaque and with results of autoradiography, and could be specifically blocked in ex vivo competition experiments [45]. These results were confirmed in a study with 46 patients with stable or unstable (recent MI) atherosclerotic lesions using ^18^F-fluciclatide for α_v_β_3_-directed PET imaging. The authors provided evidence of co-localization of radiotracer uptake with regions of increased α_v_β_3_ expression as well as with markers of inflammation and angiogenesis. Additionally, patients with unstable plaques showed a higher aortic tracer uptake than patients with stable disease [46].

In acute MI, ^18^F-fluciclatide showed increased tracer uptake in the infarcted area in a study with 21 patients, which correlated with functional recovery at follow-up but did not correlate with infarct size or C reactive protein [47]. This confirmed observations from a previous preclinical study exploring ^18^F-galacto-RGD tracer for acute MI in a rat model [48]. A recent study including 12 patients with MI that were examined 2–6 weeks post-MI with ^18^F-galacto-RGD showed that tracer uptake was more pronounced in patients with larger infarcts and was generally more intense but not restricted to areas with more impaired blood flow, proving that tracer uptake was largely independent of unspecific perfusion effects [109]. In a study with 23 MI patients and 16 stroke patients, patchy ^68^Ga-PRGD2 uptake was observed around the ischemic region in both MI and stroke patients, which was correlated with disease severity. Highest uptake was measured around 1–3 weeks after the ischemic event, whereas older or small lesions displayed no uptake [49].

In non-cardiovascular inflammatory conditions, α_v_β_3_-directed PET has mainly been used to image angiogenesis in inflammatory conditions. In a study of 20 patients with untreated RA, the authors compared ^18^F-FDG with ^68^Ga-PRGD2 PET/CT and found that ^68^Ga-PRGD2 was better suited to evaluate disease severity. They further provided histological confirmation of increased expression of α_v_β_3_ on neo-endothelial cells of the affected synovia [50].

Numerous other PET tracers targeting CAMs are currently being evaluated for inflammation imaging and cannot be extensively reviewed in this article. One of them is vascular adhesion protein-1 (VAP-1), a glycoprotein expressed on endothelial cells that is involved in transmigration of leukocytes from blood to inflamed tissues. Several promising radiolabeled antibodies and peptides targeting VAP-1 have been investigated for imaging inflammation in several conditions [110,111,112,113,114]. Similarly, selectins, Ca^2+^-dependent single-chain transmembrane glycoproteins that are classified into three subtypes depending on the cell type that expresses them (E-selectin for endothelial cells, L-selectins for leukocytes and P-selectin for platelets and endothelial cells) are interesting candidates [115]. Radiolabeled probes for imaging P-selectin by PET such as ^68^Ga-fucoidan have been investigated in several preclinical models of atherosclerosis [116,117,118].

### 2.5. Fibroblast Activation Protein-α (FAP)

Fibroblast activation protein-α (FAP) is a 170-kDa transmembrane serine protease. FAP expression is high in activated stromal fibroblasts at sites of tissue remodeling and can also be expressed in some cancer cells such as sarcoma [119]. Therefore, high levels of FAP are present in pathological conditions including fibrosis, scaring/granulation tissue, cancer, and arthritis [120]. FAP-directed PET has been introduced for imaging FAP expression of cancer and its stromal microenvironment. FAP expression is associated with a poor prognosis in several types of tumors [59,60].

Several radiolabeled antibodies have been assessed to image FAP expression in inflammatory conditions, particularly in RA. These antibodies demonstrated high tracer accumulation in inflamed joints and a correlation of radiotracer uptake with the severity of disease [61,62,63]. However, the slow clearance of FAP antibodies leads to a high background signal resulting in a limited sensitivity for detection of small lesions. This can be overcome by using radiolabeled small molecule enzyme inhibitors that have recently been developed at the University of Heidelberg and German Cancer Research Center (DKFZ) [121,122]. FAP inhibitors (FAPI) are particularly interesting since they are currently being investigated as pharmacological treatment as well as radioligand therapy to reduce fibrotic activity and FAPI PET might serve as a companion diagnostic in this setting. FAPI radioligands further provide an excellent contrast due to low FAP expression in physiological tissues.

IgG4-related disease (IgG4-RD) is characterized by an autoimmune reaction associated with fibrosis which is predominantly found in pancreas, salivary glands, kidneys, and aorta, but also other organs. Recently, two studies directly compared ^68^Ga-FAPI and ^18^F-FDG PET in the setting of IgG4-RD [64,65]. Luo and colleagues examined 26 patients with IgG4-RD using ^68^Ga-FAPI-PET/CT and detected 13% additional organ involvements in 50% of the patients compared to ^18^F-FDG-PET/CT. Furthermore, the authors detected a significantly higher FAPI compared to ^18^F-FDG uptake, which was attributed to the presence of activated fibroblasts [64]. Schmidkonz et al. studied 27 patients with inflammatory, fibrotic and overlapping manifestations of IgG4-RD using ^68^Ga-FAPI-04 and ^18^F-FDG-PET/CT as well as MRI and histology. The authors showed that the ^18^F-FDG signal mainly co-localized to IgG4^+^ cells in histology whereas the FAPI signal predominantly correlated to activated FAP^+^ fibroblasts. Of note, fibrotic lesions showed only partial reduction in their ^68^Ga-FAPI-04 PET/CT activity in response to anti-inflammatory treatment, whereas it significantly reduced ^18^F-FDG PET uptake in >90% of inflammatory lesions [65].

**Figure 3:**
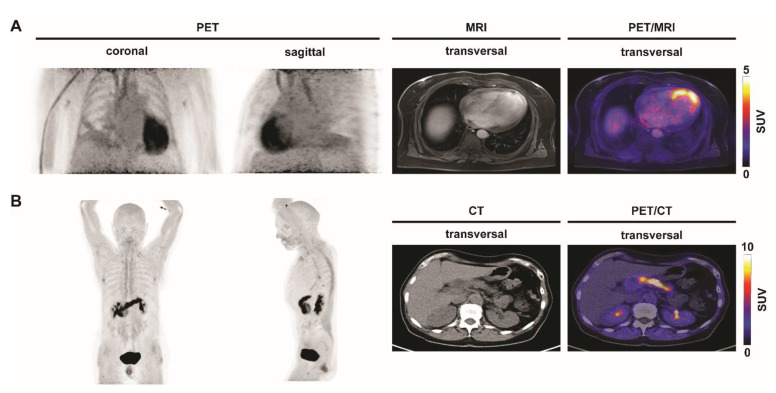
Inflammation imaging with ^68^Ga-FAPI in various inflammatory conditions. Representative coronal, sagittal and transversal ^18^F-FDG PET/CT or PET/MRI images of patients with different inflammatory conditions. (**A**) Myocardial infarction. Patient with myocardial infarction after occlusion of the left anterior descending coronary artery. ^68^Ga-FAPI-PET shows increased tracer uptake in the affected myocardium. (**B**) Chronic pancreatitis. ^68^Ga-FAPI-PET shows inflammatory activity in a patient with chronic pancreatitis. SUV, standardized uptake value.

FAP-directed PET imaging is also investigated for MI (Figure 3A). In a preclinical model of MI, Varasteh and colleagues demonstrated that ^68^Ga-FAPI-04 uptake in the injured myocardium peaked on day 6 after coronary ligation. Autoradiography and histology revealed that ^68^Ga-FAPI-04 accumulated mainly in the border zone of the infarcted myocardium [66]. Recently, Siebermair et al. retrospectively analyzed ^68^Ga-FAPI-PET images for cardiac tracer uptake of 32 patients that initially underwent PET analysis for tumor staging. The authors found an association with coronary artery disease (CAD), age, and left ventricle ejection fraction (LVEF) with FAPI uptake [67].

Despite these interesting results and further potential diagnostic applications of FAP-directed PET imaging, for instance in chronic pancreatitis (Figure 3B), fibrosis of lung, liver and kidneys, sarcoidosis, RA and possibly also atherosclerosis, studies are warranted to assess the prognostic value of FAP(I)-directed PET as well as to determine whether it can be used to risk-stratify patients in these various inflammatory and fibrotic diseases.

### 2.6. Folate Receptor

A novel, emerging target for visualizing macrophages is the folate receptor (FR), a glycosylphosphatidylinositol-anchored cell membrane protein that binds the vitamin folic acid with very high (nanomolar) affinity and internalizes it via endocytosis [123]. Over-expression of FR on cancer cells and during inflammation has been used as a diagnostic and therapeutic tool to enable targeted delivery to tumors and sites of inflammation [57,124]. The beta isoform of the folate receptor (FR-β), predominantly expressed on activated macrophages, is a promising imaging marker for inflammatory conditions [52,125].

Since FR-β has been recognized as an important transport route for methotrexate, the standard of care in RA therapy, it is not surprising that FR-β has been predominantly targeted for the evaluation of its diagnostic and therapeutic value in preclinical models of RA [51,52]. In 2013, Gent and colleagues evaluated ^18^F-fluoro-PEG-folate in a rat model of RA and compared it to the performance of the mitochondrial translocator protein (TSPO) PET tracer ^11^C-PK11195. ^18^F-fluoro-PEG-folate specifically bound to FR in blocking experiments and had a better target-to-background-ratio compared to ^11^C-PK11195 [53]. However, it is unclear whether ^18^F-fluoro-PEG-folate binds solely to the beta-isoform or to all FRs. Nonetheless, ^18^F-fluoro-PEG-folate was responsive to methotrexate treatment in other studies [51,54]. A first in-man study of six patients with RA reported fast clearance of ^18^F-fluoro-PEG-folate from heart and blood vessels, no dose limiting uptake in organs, a higher target-to-background-ratio compared to ^11^C-PK11195, and a fast uptake in affected joints already 1 min after injection of the tracer [55].

Suitability of ^18^F-fluoro-PEG-folate for imaging cardiac inflammation was established in a rat model of myocarditis by demonstrating specific radiotracer uptake in the inflamed, but not unaffected remote, myocardium; this was confirmed by autoradiography and blocking with the unlabelled FR-β ligand folate glucosamine [56]. Recently, ^68^Ga-labelled folate tracers have been developed as well [57,126]. Moisio et al. evaluated ^68^Ga-NOTA-folate (^68^Ga-FOL) in atherosclerotic mice and could show a significantly higher plaque-to-healthy vessel wall ratio compared to ^18^F-FDG PET. They further provided autoradiographic and histological evidence that ^68^Ga-FOL radioactivity co-localized with Mac3^+^ macrophage-rich atherosclerotic plaques in the aorta [57].

Another interesting, clinically evaluated, fluorine-labelled, FR-targeting tracer is 3′-Aza-2′-^18^F-fluoro-folic acid (^18^F-AzaFol) [127,128]. Schniering and colleagues showed in a preclinical proof-of-concept study that pulmonary accumulation of ^18^F-AzaFol peaked at day 7 in a mouse model of interstitial lung disease and reflected macrophage-related disease development with good correlation of folate receptor-β positivity with radiotracer uptake [58]. In a recent first-in-human multicenter clinical trial, ^18^F-AzaFol was successfully evaluated for FR targeting specificity, dosimetry and safety in cancer patients (NCT03242993) [129]. ^18^F-AzaFol PET has even been proposed as a potential tool for risk-stratification of COVID-19 patients but further clinical studies evaluating the value of ^18^F-AzaFol in inflammatory lung diseases are warranted before any conclusions can be drawn [130].

### 2.7. Mitochondrial Translocator Protein (TSPO)

Another well-characterized inflammation imaging target, especially in the field of neuroimaging, is the 18-kDa mitochondrial translocator protein (TSPO), which is up-regulated in activated microglia and systemic monocytes [131]. The transmembrane protein located in the outer mitochondrial membrane is widely distributed in most peripheral organs including kidneys, lungs, and the heart, but also nasal epithelium and adrenal glands [132]. Several PET radioligands for TSPO have been described in the past years such as ^11^C-PK11195 or ^18^F-flutriciclamide (^18^F-GE180) [133].

In a murine model of MI, elevated levels of myocardial ^18^F-GE180 uptake were observed at 1 week post-MI compared to sham-operated mice, which was localized to activated CD68^+^ inflammatory cells within the infarct area [38]. Interestingly, MI as well as subsequent heart failure was accompanied by severe neuroinflammation and TSPO PET signal was elevated in remote myocardium at 8 weeks post-MI without infiltration of inflammatory cells, suggesting a mitochondrial dysfunction in remote cardiomyocytes [38]. Moreover, TSPO signal at 1 week post-MI negatively correlated with LV ejection fraction measured at 8 weeks post-MI and the results could be confirmed in 3 patients after acute MI. Radiotracer uptake also responded to treatment with angiotensin-converting enzyme inhibitor lowering acute inflammation in the heart and brain and improved cardiac outcome [38].

Patients with atherosclerosis that underwent ^11^C-PK11195 PET combined with computed tomography angiography (CTA) displayed a high radiotracer uptake in carotid plaques. Plaques associated with recent ischemic attacks showed the highest radiotracer uptake. Of note, TSPO PET detected inflammatory activity even in asymptomatic patients and might therefore be a useful tool for early disease detection [39]. In the setting of vascular inflammation evaluated in patients with systemic inflammatory disorders, ^11^C-PK11195 PET visualized activated macrophages in the vessel wall [40,41]. Furthermore, TSPO-directed PET detected subclinical synovitis with a higher sensitivity than MRI in patients with RA [42,43,44].

In a recent multimodal preclinical study on stroke, Barca and colleagues used ^18^F-DPA-714 and ^18^F-BR-351, a radiotracer for matrix metalloproteinases (MMPs) which are expressed by numerous cells such as microglia, astrocytes, leukocytes and endothelial cells [134]. They found that acute MMP activation after stroke is preceding TSPO-dependent gliosis and that spatial distribution of MMPs and TSPO was regionally independent with only minor overlapping of the two tracers in peri-infarct regions [134].

The ability to visualize peripheral and central inflammatory networks is a beneficial property of TSPO-directed inflammation imaging. However, TSPO PET tracers have limitations, especially for the detection of peripheral inflammation, including receptor expression on multiple cell types, the presence of radiolabelled metabolites, and variability between individuals regarding the radiotracer binding affinity due to TSPO polymorphisms [103,135].

### 2.8. Other PET Tracers and Targets That Can Be Used to Study Inflammation

Numerous other PET tracers for imaging inflammation and infection cannot be extensively covered in this review, but deserve to be mentioned. As an alternative to receptor-directed imaging, radiotracers targeting increased inflammatory cell metabolism such as ^11^C-methionine have been evaluated; however, this approach is not inflammation-specific. ^11^C-methionine is a marker of amino acid transport and protein synthesis and has mainly been used in oncology [136]. However, methionine uptake is also increased in immune cells such as monocytes/macrophages, B and T cells [137]. In inflammation, ^11^C-methionine has mainly been used to image cardiac inflammation [80,138,139,140]. Another PET radiotracer that has mostly been evaluated in cancer imaging but also holds promise in imaging inflammation is 3′-deoxy-3′-18F-fluorothymidine (FLT) [141]. So far, FLT has been assessed in RA [142] and sarcoidosis [143,144]. PET imaging with sodium ^18^F-fluoride (NaF) has been extensively used to assess bone metabolism and osteogenic activity, but can also be used as a marker of calcification activity and has been investigated in a range of cardiovascular disorders including aortic stenosis or atherosclerosis, and other inflammatory conditions, such as RA [145,146,147,148,149]. The P2 × 7 receptor, a purinergic receptor, is an ion channel expressed mainly on macrophages and monocytes as well as microglia and astrocytes. Because of its role as a key regulatory element of the inflammasome complex it has attracted some attention as an imaging target for inflammation. Various PET tracers have been assessed, mainly in the setting of neuroinflammation [150,151,152,153]. Antibody-based PET tracers have been extensively studied for their application in inflammatory diseases targeting immune cells, such as T lymphocytes (CD3 and CD4), B lymphocytes (CD20), or granulocytes (BW250/183) [154,155]. Various groups have also developed radiolabelled nanoparticles that are phagocytosed by macrophages or that are targeted towards T lymphocytes which can be used for inflammation imaging [156,157,158,159]. An interesting theranostic approach is the labelling of liposomal glucocorticoids with zirconium-89 which could be interesting for RA but also for inflammatory bowel disease [160]. For a nicely-written and comprehensive overview over the most promising candidates for infection-specific PET imaging, please refer to the review by Auletta and colleagues [161].

## 3. Conclusions

**Figure 4:**
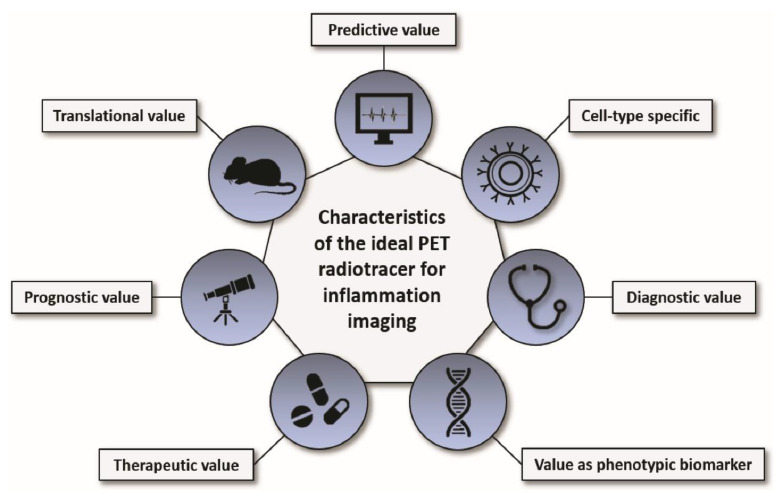
Characteristics of the ideal PET radiotracer for imaging inflammation. The perfect PET radiotracer for imaging inflammation has an excellent predictive value, is cell-type specific, shows a good target-to-background ratio (diagnostic value), has value as a phenotypic biomarker, responds to anti-inflammatory therapy (therapeutic value), has a good correlation with the functional outcome and/or progression of the disease (prognostic value), and is safe for its translation into patients (translational value).

Inflammation plays a fundamental role in many medical conditions, but restrained or excessive inflammation can have detrimental effects that can worsen the outcome of patients. Molecular imaging of inflammation has emerged as a helpful tool to non-invasively visualize and study inflammation in vivo in a variety of diseases; it shows value as a strong clinical and preclinical research application and may provide insight into the individual biology of inflammation which can have diagnostic, therapeutic, and prognostic value. The perfect PET radiotracer for inflammation imaging has an excellent predictive value, is cell-type specific, shows a good target-to-background ratio (diagnostic value), has a value as phenotypic biomarker, responds to anti-inflammatory therapy (therapeutic value), has a good correlation with the functional outcome and/or progression of the disease (prognostic value), and is safe for its translation into patients (translational value; Figure 4). Despite promising preclinical and clinical results, none of the herein discussed radiotracers unites all of these desired characteristics, and several obstacles still need to be overcome to establish inflammation imaging in a routine clinical setting and for validated research. Improvement of PET radiotracers for imaging inflammation, accurate and standardized quantification of radiotracer uptake for interpretation and comparability of the results, comparable and reproducible imaging protocols and guidelines, further improvement of spatial resolution of PET devices (particularly important for inflammation imaging of small structures such as vessels), and a broader access to PET imaging facilities for physicians from different medical fields are just a few of the challenges that the community needs to address in the near future. Nonetheless, PET inflammation imaging may provide insight into the individual biology of inflammation which can be of great diagnostic, therapeutic, and prognostic value for patients.

## Figures and Tables

**Table 1:** Overview of radiotracers and their molecular targets for PET inflammation imaging.

Target	PET Radiotracer	Cell Types Targeted by the Radiotracer	Evaluated Diseases	Advantages	Limitations	Approved for Use in Humans?
Glucose metabolism (predominantly glucose transporter 1 and 3 (GLUT1-3))	^18^F-FDG	High-glucose-using cells such as immune cells, cancer cells, cardiomyocytes, neurons, brown adipocytes, kidney cells	Myocardial infarction [1], cancer [2], atherosclerosis [3], sarcoidosis [4], endocarditis [5], IgG4-rel. diseases [6], arthritis [7], infection and others [8]	High sensitivity, fast technique completed in one session, broad availability [8]	High background signal, often need for non- physiological suppression techniques, not inflammation-specific, limited use in some clinical settings	Yes
Mannose receptor	^18^F-FDM, ^68^Ga-NOTA-MSA, ^68^Ga-NOTA-anti-CD206 nanobody	Mainly expressed by macrophages (M2 > M1), immature dendritic cells, and liver sinusoidal endothelial cells	Mainly atherosclerosis [9,10,11] and cancer [12]	Higher cell specificity than ^18^F-FDG (M2 > M1 macrophages)	Correlation of mannose-directed PET signals with histology of leukocytes and distinction from ^18^F-FDG signal remains to be determined	No
Somatostatin receptors (SSTR)	^68^Ga-DOTATOC, ^68^Ga- DOTATATE, ^68^Ga- DOTANOC	Overexpressed mainly on pro-inflammatory M1 macrophages	Atherosclerosis [13,14], sarcoidosis [15,16,17], other sources of myocardial inflammation (i.e., pericarditis, myocarditis, MI) [18], and others [19] (i.e., idiopathic pulmonary fibrosis, histiocytosis, tuberculosis, cardiac allograft rejection, and small vessel vasculitis)	higher cell specificity and improved signal-to-background-ratio of DOTA-peptides compared to ^18^F-FDG imaging (in particular advantageous for cardiac inflammation imaging)	Often labelled with gallium-68 (need for on-site generator)	Yes
C-X-C motif chemokine receptor 4 (CXCR4)	^68^Ga-pentixafor, ^64^Cu-DOTA-FC131	Expressed on several pro-inflammatory immune cells, particularly overexpressed on macrophages and T cells	Cancer [20], atherosclerosis [21,22,23,24,25], myocardial infarction [26,27,28,29,30,31], osteomyelitis [32], urinary tract infections [33] and others	Potential theranostic target in atherosclerosis and MI; superiority over ^18^F-FDG in atherosclerosis; superior in chronic bone infections over granulocyte-directed ^99m^Tc-besilesomab and ^99m^Tc-labelled leukocytes	Not yet clinically approved, larger clinical trials needed to determine prognostic and diagnostic value in different inflammatory conditions; unspecific cellular source as various inflammatory cells express CXCR4	No; several early phase I clinical trials for cancer imaging ongoing (i.e., NCT04504526)
C-C motif chemokine receptor 2 (CCR2)	^68^Ga/^64^Cu-DOTA-ECL1i	Mainly expressed on pro-inflammatory monocytes, natural killer cells and T cells	Lung inflammation [34], cardiac injury [35], abdominal aortic aneurysm [36], pulmonary fibrosis [37]	Promising results regarding prognostic and therapy-monitoring abilities	unspecific cellular source as various inflammatory cells express CCR2; toxicity and biodistribution still need to be examined for a safe translation into the clinics	No; several phase I clinical trials ongoing, i.e., for imaging atherosclerosis (NCT04537403) and lung inflammation (NCT03492762)
Mitochondrial translocator protein (TSPO)	^11^C-PK11195 and 2nd and 3rd generation TSPO tracers, such as ^18^F-flutriciclamide (^18^F-GE180) or ^18^F-DPA-714	Protein located in the outer mitochondrial membrane; upregulated in activated macrophages, particularly in microglia	Myocardial infarction [38], atherosclerosis [39], vascular inflammation [40,41], rheumatoid arthritis [42,43,44]	ability to visualize peripheral and central inflammatory networks; superiority over MRI regarding detection of subclinical synovitis	Limited use in detection of peripheral inflammation; multicellular receptor expression profile; presence of radiolabelled metabolites; variability between individuals regarding tracer binding affinity due to TSPO polymorphisms	No; several clinical trials are ongoing especially in the field of neuroinflammation (NCT03457493, NCT04412187, NCT03662750 and others)
α_v_β_3_ integrin receptor	^18^F-galacto-RGD, ^68^Ga-PRGD2, ^18^F-fluciclatide	Mediates cell adhesion; important role in angiogenesis, expressed on a variety of cells such as activated endothelial cells, solid tumor cells, immune cells	Atherosclerosis [45,46], myocardial infarction [47,48,49], rheumatoid arthritis [50]	Superiority over ^18^F-FDG regarding evaluation of disease severity in rheumatoid arthritis (^68^Ga-PRGD2)	Not yet clinically approved, larger clinical trials needed to determine prognostic and diagnostic value in different inflammatory conditions; unspecific cellular source as various cell types express integrins	No; clinical trials have been conducted in rheumatoid arthritis (NCT01940926) and MI (NCT01813045)
Folate receptor (FR) (in particular the beta isoform (FR-β))	^18^F-Fluoro-PEG-folate;^18^F-AzaFol; ^68^Ga-Ga-NOTA-folate (^68^Ga-FOL)	High expression on cancer cells and activated M1-macrophages (and monocytes) with restricted FR expression in normal tissues	Rheumatoid arthritis [51,52,53,54,55], myocarditis [56], atherosclerosis [57], interstitial lung disease [58]	Important transport route for methotrexate making it an interesting target for rheumatoid arthritis; better target-to-background-ratio of ^18^F-fluoro-PEG-folate as compared to ^11^C-PK11195; significantly higher plaque-to-healthy vessel wall ratio of ^68^Ga-FOL as compared to ^18^F-FDG PET	Not yet clinically approved, larger clinical trials needed to determine prognostic and diagnostic value in different inflammatory conditions;	No; clinical trial for ^18^F-AzaFol in cancer imaging has been conducted (NCT03242993)
Fibroblast activation protein-α (FAP)	Various, mainly ^68^Ga-labelled FAP inhibitors such as ^68^Ga-FAPI-04; labelled antibodies directed to FAP	Fibroblasts and tumor cells	Cancer [59,60], rheumatoid arthritis [61,62,63], IgG4-related disease [64,65], myocardial infarction [66,67]	Excellent contrast due to due to low FAP expression in physiological tissues; theranostic properties since mainly FAP inhibitors are used; superiority over ^18^F-FDG in IgG4-rel. disease	Further studies are warranted to assess the prognostic and theranostic value	No; several clinical trials are ongoing, i.e., for rheumatoid arthritis(NCT04514614), IgG4-rel. disease (NCT04125511) and inflammatory bowel disease (NCT04507932)
